# Glycogen Synthase Kinase 3beta Contributes to Proliferation of Arterial Smooth Muscle Cells in Pulmonary Hypertension

**DOI:** 10.1371/journal.pone.0018883

**Published:** 2011-04-18

**Authors:** Piotr Sklepkiewicz, Ralph Theo Schermuly, Xia Tian, Hossein Ardeschir Ghofrani, Norbert Weissmann, Daniel Sedding, Tarek Kashour, Werner Seeger, Friedrich Grimminger, Soni Savai Pullamsetti

**Affiliations:** 1 Medical Clinic II/V, University Hospital, Giessen, Germany; 2 Max-Planck-Institute for Heart and Lung Research, Bad Nauheim, Germany; 3 Medical Clinic I, University Hospital, Giessen, Germany; 4 King Fahad Medical City, Riyadh, Saudi Arabia; Katholieke Universiteit Leuven, Belgium

## Abstract

**Rationale:**

Pulmonary arterial hypertension (PAH) is a rare progressive pulmonary vascular disorder associated with vascular remodeling and right heart failure. Vascular remodeling involves numerous signaling cascades governing pulmonary arterial smooth muscle cell (PASMC) proliferation, migration and differentiation. Glycogen synthase kinase 3beta (GSK3ß) is a serine/threonine kinase and can act as a downstream regulatory switch for numerous signaling pathways. Hence, we hypothesized that GSK3ß plays a crucial role in pulmonary vascular remodeling.

**Methods:**

All experiments were done with lung tissue or isolated PASMCs in a well-established monocrotaline (MCT)-induced PAH rat model. The mRNA expression of Wnt ligands (Wnt1, Wnt3a, Wnt5a), upstream Wnt signaling regulator genes (Frizzled Receptors 1, 2 and secreted Frizzled related protein sFRP-1) and canonical Wnt intracellular effectors (GSK3ß, Axin1) were assessed by real-time polymerase chain reaction and protein levels of GSK3ß, phospho-GSK3ß (ser 9) by western blotting and localization by immunohistochemistry. The role of GSK3ß in PASMCs proliferation was assessed by overexpression of wild-type GSK3ß (WT) and constitutively active GSK3ß S9A by [^3^H]-thymidine incorporation assay.

**Results:**

Increased levels of total and phosphorylated GSK3ß (inhibitory phosphorylation) were observed in lungs and PASMCs isolated from MCT-induced PAH rats compared to controls. Further, stimulation of MCT-PASMCs with growth factors induced GSK3ß inactivation. Most importantly, treatment with the PDGFR inhibitor, Imatinib, attenuated PDGF-BB and FCS induced GSK3ß phosphorylation. Increased expression of GSK3ß observed in lungs and PASMC isolated from MCT-induced PAH rats was confirmed to be clinically relevant as the same observation was identified in human iPAH lung explants. Overexpression of GSK3ß significantly increased MCT-PASMCs proliferation by regulating ERK phosphorylation. Constitutive activation of GSK3ß (GSK3ß S9A, 9th serine replaced to alanine) inhibited MCT-PASMCs proliferation by decreasing ERK phosphorylation.

**Conclusion:**

This study supports a central role for GSK3ß in vascular remodeling processes and suggests a novel therapeutic opportunity for the treatment of PAH.

## Introduction

Pulmonary arterial hypertension (PAH) is a progressive pulmonary vascular disorder with high morbidity and mortality [1], [2]. The pulmonary vascular remodeling may typically involve numerous molecular signaling cascades governing endothelial dysfunction, neovascularization of small pulmonary arteries, pulmonary arterial smooth muscle cell (PASMC) and adventitial fibroblast (PAAF) migration and proliferation [3], [4]. However, 3 of the currently approved therapies targeting vasoconstrictive/vasodilatory abnormalities in PAH, like endothelin, nitric oxide or prostacyclin, show beneficial effects and improved quality of life [5], [6], [7], but do not appear to reverse or modify disease progression.

To this end, in the last 5 years, enormous progress has been made by our group and authors in developing new anti-proliferative and pro-apoptotic therapeutic strategies to reverse the disease progression. The role of growth factors, tyrosine/serine-threonine kinase receptors in PAH has been extensively studied at the cellular, preclinical and clinical levels. Accumulation of growth factors such as platelet derived growth factor (PDGF), epidermal growth factor (EGF), fibroblast growth factor (FGF) and vascular endothelial growth factor (VEGF) and pro-survival factors like survivin in the pulmonary vasculature suggest categorizing this disease as a pseudo-malignant proliferative disorder [8], [9], [10], [11], [12]. In accordance, inhibition of anti-cancer compounds, Imatinib/STI571 (PDGFRß, C-kit, SDF-1), PKI166 (EGFR) and Sorafenib (multikinase inhibitor), prove effective in reversing vascular remodeling and improved survival in various experimental models of PAH [8], [10], [13]. These studies not only highlighted the importance of anti-cancer therapeutics in PAH but also open avenues to explore new intracellular signaling pathways in the PAH disease pathogenesis.

GSK3ß (Glycogen Synthase Kinase-3 beta) is a ubiquitously expressed, highly conserved serine/threonine protein kinase found in all eukaryotes. Although initially identified as a regulator of glycogen metabolism, GSK3ß can act as a downstream regulatory switch for numerous signaling pathways [14], [15]. GSK3ß is constitutively active in unstimulated, resting cells, and is inactivated during cellular responses. Treatment of cells with a growth factors such as insulin, is shown to cause GSK3ß inactivation through a PI 3-kinase (PI3K)-dependent mechanism. PI3K-induced activation of PKB/Akt results in phosphorylation of ser 9 on GSK3ß, that inhibits GSK3ß activity [16], [17]. Similarly, GSK3ß was shown to play a key inhibitory role in the Wnt signaling pathway [18]. This inactivation causes reduced phosphorylation of its substrates, such as ß-Catenin, c-Myc, endothelin, VEGF, survivin etc [19], [20], [21]. Thus, GSK3 by regulating several of its substrates participates in a wide spectrum of cellular processes, including glycogen metabolism, transcription, translation, cytoskeleton regulation, intracellular vesicular transport, cell cycle progression and apoptosis.

Recent evidences suggest that inactivation of GSK3ß can positively regulate proliferation and cell survival in many types of cancer and in vascular remodeling [22], [23]. Hence we hypothesized that GSK3ß plays a crucial role in remodeling of vascular components during PAH. Our aims were at first to determinate expression profile of GSK3ß and its regulatory genes (Wnt ligands 1, 3a, 5a, Frizzled Receptor 1 and 2, sFRP-1 and Axin1) and quantify protein level changes and phosphorylation status in MCT-induced PAH rat lungs and in isolated PASMCs. Further, to assess the functional role of GSK3ß in PASMCs proliferation and down stream molecular mechanisms by overexpression of wild type and constitutively active mutant of GSK3ß.

## Materials and Methods

### Ethics Statement

The study protocol for human tissue donation was approved by the ethics committee (Ethik Kommission am Fachbereich Humanmedizin der Justus Liebig Universität Giessen) of the University Hospital Giessen (Giessen, Germany) in accordance with national law and with “Good Clinical Practice/International Conference on Harmonisation” guidelines (AZ 31/93). A written informed consent was obtained from each individual patient or the patient's next to kin.

The University Animal Care Committee and the Federal Authorities approved all animal studies for Animal Research of the Regierungspräsidium Giessen (Hessen, Germany) (Az. GI 20/10 Nr. 09/2010).

### Materials

GSK3ß total, phospho-GSK3ß (ser 9), AKT, phospho-AKT antibodies were purchased from Cell Signaling (Denver, USA). Axin1, phospho-ERK1/2, ERK total and H1 histone antibodies were obtained from Santa Cruz (Heidelberg, Germany). GAPDH antibody was obtained from Novus (Littleton, USA). Dulbecco's Modified Eagle's medium, nutrient mixture F-12 (DMEM-F12), fetal calf serum (FCS), Streptomycin/Penicillin, Vitamins and non-essentials amino acids were obtained from Gibco (Karlsruhe, Germany). Platelet derived growth factor BB (PDGF-BB) was purchased from PeproTech (Hamburg, Germany). Wnt3A ligand was purchased from R&D Systems (Minneapolis, USA). PDGFRß inhibitor Imatinib (Gleevec) was obtained from Novartis (Switzerland). Im Prom reverse transcriptase and Taq polymerase PCR Kit were obtained from Promega (Mannheim, Germany). RIPA buffer was obtained from Santa Cruz (Heidelberg, Germany). ECL detection kit was obtained from Amersham Biosciences (Piscataway, USA). Histostain SP Rabbit Primary (AEC) was purchased from ZYMED Laboratories (Carlsbad, USA). MiniPrep Plasmid Isolation Kit was obtained from PeqLab (Erlangen, Germany) and MaxiPrep Plasmid Isolation Kit, PCR purification Kit was purchased from QIAGEN (Hilden, Germany), pGEMT-Easy vector Kit was obtained from Promega, (Mannheim, Germany), pcDNA3.1 Directional TOPO Expression Kit, Lipofectamine 2000, T4 Ligase, Platinum Taq DNA Polymerase High Fidelity, Platinum SYBR Green qPCR SuperMix-UDG was purchased from Invitrogen (Karlsruhe, Germany), restriction enzymes (BamHI, BsmI, EcoRV) was purchased from New England BioLabs (Frankfurt, Germany). Gel Extraction Kit was obtained from QIAGEN (Hilden, Germany), Opti-DMEM medium was purchased from Gibco (Karlsruhe, Germany).

### Animal experiments

Experiments were performed on male CD rats of 300–350 g body weight (Charles River, Sulzfeld, Germany). Pulmonary arterial hypertension was induced by a single subcutaneous injection of monocrotaline (MCT, 60 mg/kg, Sigma, Deishofen, Germany), dissolved in 0.1 M NaOH, adjusted to pH 7.4 with 0.1 M HCl, as described previously [24]. In these experiments 3 study groups were used: healthy rats, MCT-injected rats sacrificed either 3 weeks or 5 weeks after injection.

### Pulmonary arterial smooth muscle cells isolation

Rat PASMCs were isolated from pulmonary arteries using the explant method as described previously [8] and the characterization and purity was assessed as described [25]. Briefly, characterization of PASMCs was done using immunocytochemical staining for a-smooth muscle actin and desmin. The presence of endothelial cells and fibroblasts were excluded by staining for CD31 and FSP1. Further in order to determine the purity of PASMCs, flow cytometric analysis was performed with anti-desmin and anti-α-smooth muscle actin antibodies. Usually, the purity of isolated PASMCs is 91–94%. Cultures were maintained at 37°C in a humidified 5% CO_2_/95% O_2_ atmosphere. All experiments using PASMCs were performed between 2–4 passages. Expression analysis studies were done at early passages to minimize the influence of phenotypic alterations.

### Cell culture

Primary rat PASMC's were maintained at 37°C in a humidified 5%CO_2_/95%O_2_ atmosphere. Cells were grown on 10 cm^2^ dishes, 6 and 48-well plates in DMEM-F12 supplemented with 10% FCS, 5% streptomycin/penicillin, 5% glutamate. Cells were cultured from the time of isolation in 10% FCS medium and subsequently in experiments with 0.1% FCS medium supplemented with PDGF-BB (60 ng/ml) or Wnt3a (100 ng/ml) for 6 and 24 hrs. Additionally, PASMC's were stimulated with PDGF-BB (60 ng/ml) for 6 hrs and 24 hrs and were also pretreated with 1 and 5 µM of the PDGFRβ inhibitor Imatinib (Gleevec) in the presence of 10% FCS.

### Reverse transcriptase PCR and Real-Time PCR

Total RNA was isolated from tissue rat's lung homogenates and primary rat pulmonary arterial smooth muscle cells (PASMC) with Trizol® Reagent. The quantity and quality of RNA was determined by NanoDrop (PeqLab, Erlangen, Germany). Equal amounts of RNA from each sample were used as templates for reverse transcription reaction for generation of cDNA using Im Prom Reverse Transcriptase and Taq polymerase PCR Kit with oligo(dT)_18_ primers according to the supplier's instructions. Later, quantitative Real-Time PCR analysis was performed as described previously [26]. Briefly, 1 µl cDNA was placed into 25 µl reaction volume containing Platinum SYBR Green qPCR SuperMix-UDG and sequence-specific oligonucleotide primers. The thermal cycle conditions used for all reactions were as follows: activation, 50°C for 2 min; denaturation, 95°C for 10 min; and cycle, 95°C for 10 s and 59°C for 30 s (40 times). Specific primers used for sequence detection both in tissue homogenates as well as in the cells were: Wnt1 (Forward 5′CTA CGT TGC TAC TGG CAC TGA C3′, Reverse 5′AGA CTC TTG GAA TCT GTC AGC AG3′), Wnt3a (Forward 5′ATT TGG AGG AAT GGT CTC TCG3′, Reverse 5′GCA GGT CTT CAC TTC GCA AC3′), Wnt5a (Forward 5′GCC ACT TGT ATC AGG ACC ACA3′, Reverse 5′GGC ATT TAC CAC TCC AGC AG3′), sFRP-1 (Forward 5′GCT AGA GAG GAG CCC TGA AAA T3′, Reverse 5′TGC ACT GTA TCC CTC TAT CTT GC3′), Frizzled 1 receptor (Forward 5′CGT ACT GAG TGG AGT GTG TTT TG3′, Reverse 5′TGA GCT TTT CCA GTT TCT CTG TC3′), Frizzled 2 receptor (Forward 5′GTG TAG AGC ACG GAG AAG ACG3′, Reverse 5′TAC CTG TTC ATC GGC ACA TC3′), Axin1 (Forward 5′CCA CAG AAA TAG TAG GCC ACA3′, Reverse 5′GGA GGA AGA AGA AAA GAG AGC3′), GSK3ß (Forward 5′TCG CCA CTC GAG TAG AAG AAA3′, Reverse 5′ACT TTG TGA CTC AGG AGA ACT3′), PBGD (Forward 5′CAA GGT TTT CAG CAT CGC TAC3′, Reverse 5′ATG TCC GGT AAC GGC GGC3′).

### Protein isolation and Western blotting

Lung tissue and PASMC's samples were homogenized using tissue homogenizer (for tissues only) or lysed in RIPA buffer (Santa Cruz) with addition of a protease inhibitor cocktail and PMSF. Tissue and cells lysates were equalized with SDS 5× sample buffer and electrophoretically separated on 10% polyacrylamide gels and transferred for 1 h on to nitrocellulose membranes. Subsequently membranes were blocked 1 h with 5% non-fat dry milk in Tris-buffered saline/0.1% Tween 20. After blocking, membranes were probed with primary antibodies diluted as follows: GSK3ß total (1∶1000), phospho-GSK3ß (ser 9) (1∶1000), AKT (1∶1000), phospho-AKT (1∶1000), Axin1 (1∶500), phospho-ERK1/2 (1∶1000), ERK total (1∶1000), GAPDH (1∶5000). Samples were normalized to GAPDH housekeeping gene and total protein of interest (when examining protein phosphorylation) and further quantification was performed. After primary antibody incubation, membranes were incubated with secondary goat anti-rabbit (1∶30000) or rat anti-mouse (1∶50000) HRP-conjugated antibodies (Sigma). Signals were then detected by ECL detection system and further quantified using specific software as described [27].

### Immunohistochemistry

Paraffin-embedded lung sections were cut to 3 µm thickness by microtome and incubated 45 minutes at 65°C and subsequently deparaffinized in xylene three times, each 3 minutes. Samples were hydrated with 100% to 80% ethanol concentration washed with water and the antigen was unmasked by microwaving 20 minutes in the retrieval buffer. Sections were incubated in 3% hydrogen peroxide in methanol for 20 minutes at room temperature to block endogenous peroxidase activity, washed and blocked for 30 minutes in 2% bovine serum albumin. Then sections were probed overnight at 4°C with primary anti-rabbit antibody (GSK3β Cell Signaling, 1∶50 dilution). After overnight incubation sections was washed with PBS and incubated with secondary biotinylated horse anti-rabbit IgG Antibody (Vector Laboratories) for 30 min, followed by incubation with the ABC reagent kit (avidin and biotinylated peroxidase, Vector Laboratories) for 30 min. Development of the target-bound peroxidase for detection of GSK3β was carried out with Vector NovaRed substrate kit for peroxidase according to manufacturer's instruction (Vector Laboratories). Finally, sections were counterstained with haematoxylin (Zymed Laboratory, Cambridge, UK), mounted with VectaMount (Vector Laboratories) and visualized by light microscopy (bright-field microscopy).

### Cloning

Human GSK3ß wild type full length insert was obtained on the human PASMCs cDNA template by PCR reaction using Platinum Taq DNA Polymerase High Fidelity with the following primers: Forward 5′CCT AAC ACC CCA ACA TAA AGA CA3′, Reverse 5′GTA ACT GGT GGT TTT TCC TGT GC3′. Human GSK3ß WT insert was subsequently ligated into the pGEMT-Easy Vector and then electroporated to the DH5α competent strain of *E.Coli*, becoming the template for following active GSK3ß mutant construction. Later GSK3ß full length and mutant constructs was transferred to mammalian pcDNA3.1 expression vector TOPO cloning system and transformed to JM-109 compentent *E.Coli* strain following heat shock procedure. After transformation constructs were digested with *Bam*HI and verified on a 0.7% agarose gel. Pure plasmid GSK3ß constructs were obtained using MaxiPrep Plasmid Isolation Kit in extensive amounts for subsequent transient transfection and further sent for sequencing to AGOWA GmbH sequencing service (Berlin, Germany).

### Mutagenesis and Sequencing

Wild type pGEMT- GSK3ß was used as a template for creating mutants of GSK3ß which are S9A- constitutively active mutant of GSK3ß where serine 9 residue was substituted with alanine. To prepare constitutively active constructs of GSK3ß, point mutations were done with long template PCR technique with use of following mutagenesis primers: GSK3ß S9A (Sense 5′GGC CCA GAA CCA CCG CAT TCG CGG AGA GCT GCA A3′; Antisense 5′TTG CAG CTC TCC GCG AAT GCG GTG GTT CTG GGC C3′) in the following set up (95°C-90 s; 95°C-30 s; 62°C-30 s; 68°C-900 s; 68°C-1200 s; 4°C). Digestion of methylated template was done by 3 h incubation in 37°C with use of *Dpn*I enzyme. Subsequently precipitated plasmid was transformed to JM-109 strain of *E.Coli* and produced in extensive amounts for subsequent transfections.

### Transient transfection of PASMCs with pcDNA3.1 TOPO-GSK3β construct

PASMCs were plated in 6 or 48-well plates until they reach 60 to 80% confluence at the time of transfection. Empty pcDNA3.1 vector and pcDNA3.1-GSK3ß construct (GSK3ß WT, S9A) were mixed with Opti-DMEM F12 medium and combined with Lipofectamine 2000 transfection reagent and added to cultured MCT PASMCs for 6 hrs as described [28]. After this time cells were washed with PBS and stimulated with 10% FCS to allow cell recovery for next 18 hrs and later depending on further assays adequately stimulated with 0.1 or 10% FCS supplied DMEM F12 medium.

### Proliferation Assay

For assessment of proliferation, rat PASMCs from passage 2 were seeded in 48-well plates. Primary cells were starved by incubation for 24 hrs in DMEM containing 0.1% FCS. Subsequently, they were stimulated with 10% FCS/DMEM or PDGF-BB (60 ng/ml) to induce cell cycle reentry. During the last 4 hrs of the stimulation period, cells were pulsed with 1.5 µCi per well [^3^H] thymidine (Amersham Pharmacia Biotech Ltd). The [^3^H] thymidine content of cell lysates was determined by scintillation counting as described previously [8]. Data were expressed as counts per minute (cpm) and normalized to the amount of cells per well. In our study all of [3H] thymidine uptake experiments were carried out on 48-well plates and the amount of PASMCs seeded on one well was around 30,000.

### Patient characteristics

Human lung tissue was obtained from donors and iPAH patients undergoing lung transplantation. Donor lung tissue was from non-transplanted lung tissue of transplant donors. Donor lungs were explanted according to a standard European explant protocol (Eurotransplant), using cold perfused with preservation buffer and stored inflated on ice until use. Non-transplanted donor lung was not transplanted because i) the complete lung would not fit into the recipient thorax and a part was resected or ii) due to on site decision of the transplant surgeon not to use the lung, based on edema, pulmonary thrombi or obvious pneumonia. In either case, lung tissue was snap-frozen directly after transplantation.

## Results

### GSK3β is dysregulated in total lung homogenates of monocrotaline-induced pulmonary arterial hypertension in rats

**Figure 1:**
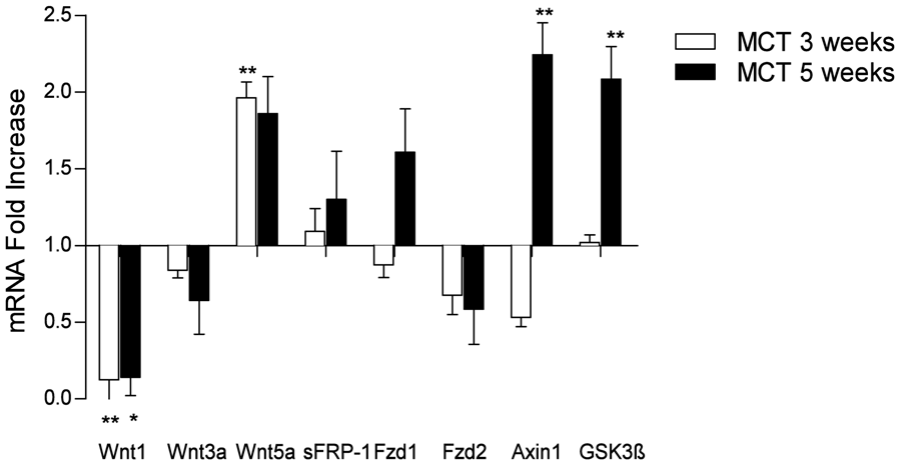
Expression of Wnt signaling upstream regulators of GSK3β in lung tissues of control and MCT-induced PAH rats. mRNA expression of Wnt1, Wnt3a, Wnt5a, Frizzled 1, Frizzled 2, sFRP-1, Axin 1 and GSK3β in lung homogenates from control and after 3 weeks (white bar) and 5 weeks (black bar) of MCT-induced PAH rats, as analyzed by quantitative real-time PCR. All values were given as the mean ± SEM (n = 3) and were normalized to Porphobilinogen deaminase (PBGD). Values were presented significant as *P<0.05, **P<0.01 *vs* control lungs. Healthy controls were set as 1 on X axis and expression profile from 3 and 5 weeks MCT were presented as fold of gene regulation.

**Figure 2:**
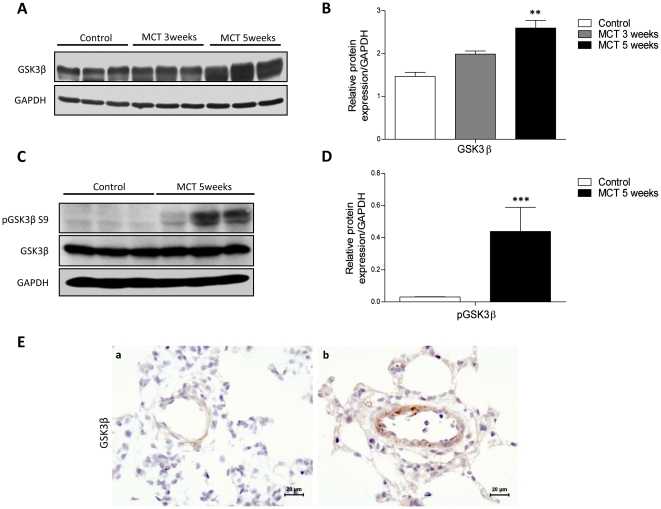
Increased GSK3ß and its phosphorylated form in MCT-induced PAH rat lungs. Protein expression as analyzed by (**A**) western blotting and subsequent (**B**) densitometric quantification of GSK3ß in control (white bar) and after 3 weeks (grey bar) and 5 weeks (black bar) of MCT-induced PAH in rats. Phosphorylation analysis by (**C**) western blotting and subsequent (**D**) densitometric quantification of pGSK3ß (serine 9) in control (white bar) and 5 weeks (black bar) lungs of MCT-induced PAH in rats. GAPDH was used as a loading control. Values were presented significant as **P<0.01, ***P<0.001 *vs* control lungs. All values were expressed as mean ± SEM (n = 3). (**E**) Immunohistochemical localization of GSK3β in the healthy lungs (a) and lungs 5 weeks after MCT injury (b). Magnification 40×.

Expression of Wnt ligands (Wnt1, Wnt3a, and Wnt5a) and Wnt signaling upstream regulator genes (Frizzled1 and 2 receptors and sFRP-1) and intracellular effectors (Axin1 and GSK3β) were investigated in pulmonary hypertensive rat lungs, 3 and 5 weeks after MCT injury. Real-time RT-PCR demonstrated a decrease in Wnt canonical ligands (Wnt1 and Wnt3a) with no significant changes in Frizzled receptors expression in 5 weeks of MCT-PAH rat lungs (Figure 1). Parallel increase in mRNA expression of GSK3ß and Axin1 was observed (Figure 1). Western blotting demonstrated a significant increase in total GSK3ß expression in MCT lungs 3 and 5 weeks post MCT injury (Figure 2A, B) as well as significant increase in its phosphorylation in 5 weeks MCT-PAH rat lungs (Figure 2C, D). However, phosphorylation of GSK3ß was not altered 3 weeks after MCT treatment (data not shown). Immunohistochemical analysis suggested an immunoreactivity of GSK3ß was observed in the medial layer of pulmonary arteries of both 5 weeks MCT-PAH rat lungs and control PAH rat lungs (Figure 2E).

### Increased GSK3ß and it's phosphorylated form in pulmonary arterial smooth muscle cells isolated from MCT-PAH rats

**Figure 3:**
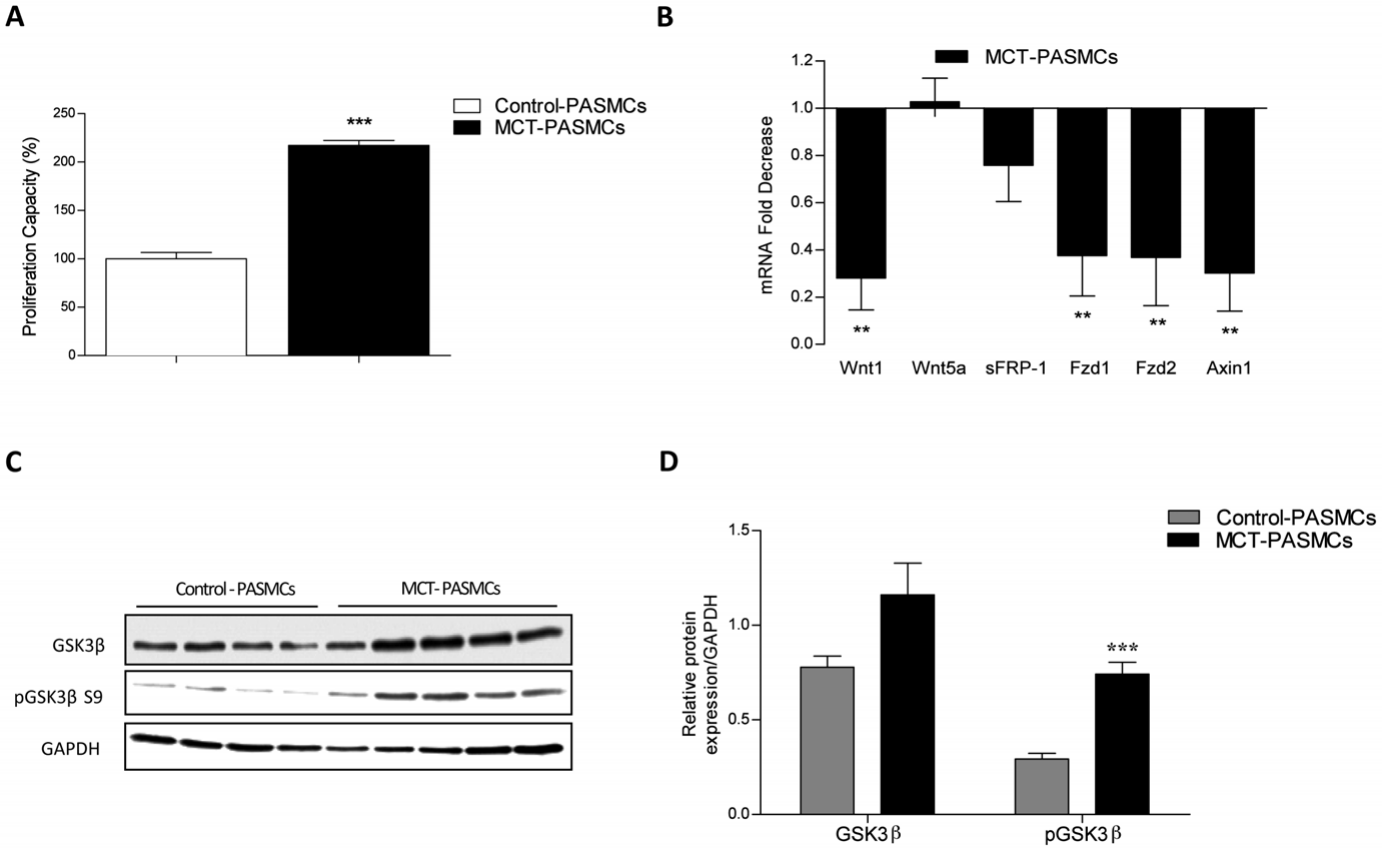
Increased GSK3ß and its phosphorylated form in primary PASMCs isolated from lungs of control and MCT-induced PAH rats. (**A**) Proliferation capacity of primary rat MCT-PASMCs compared to healthy control-PASMCs isolated from rat lungs 5 weeks post MCT injury in 10% FCS conditioned media was assessed by [3H]-thymidine incorporation (n = 5). Data were obtained as counts per minute (cpm) and normalized to the amount of cells per well. All values were expressed as the percentage of proliferation capacity (mean ± SEM). Values were presented significant as *** P<0.001 *vs* control. (**B**) mRNA expression of Wnt1, Wnt3a (not expressed), Wnt5a, Frizzled 1, Frizzled 2, sFRP-1, Axin 1 and GSK3ß in primary control PASMCs and PASMCs from MCT-induced PAH rats isolated from the lungs 5 weeks after MCT injection, as analyzed by quantitative real-time PCR. Rat PASMC were maintained in culture media supplemented with 10% FCS. All values were normalized to Porphobilinogen deaminase (PBGD) and were presented as fold of gene regulation with a control set as 1. Values were presented significant as **P<0.01 *vs* control PASMCs. All values were expressed as mean ± SEM (n = 4). Protein expression as analyzed by (**C**) western blotting and subsequent (**D**) densitometric quantification of total GSK3ß/GAPDH, phosphorylation of GSK3ß at serine 9 residue (pGSK3ß S9/total GSK3ß) in primary PASMCs isolated from control (grey bar) and MCT-induced PAH rats (black bar). GAPDH was used as a loading control. All values were expressed as mean ± SEM (Control PASMCs, n = 4; MCT-PASMCs n = 5). Values were presented significant as ***P<0.001 *vs* control PASMCs. Both mRNA and protein were isolated from healthy control- and MCT-PASMCs at passage 3 in 10% FCS conditioned media.

Interestingly PASMCs isolated from the rat lungs 5 weeks after MCT injury displays much stronger proliferative phenotype as compared to healthy control-PASMCs in 10% FCS condition (Figure 3A) and PDGF-BB (60 ng/ml) supplemented media (Figure S1). Therefore, we tested the expression profile of GSK3ß-related genes (Figure 3B) in these hyper-proliferative PASMCs. Similarly to the mRNA level in the lungs after MCT injury we found a decrease in canonical Wnt1 (Figure 3B). On the other hand, reduction in Frizzled receptor 2 is significant in isolated MCT-PASMCs, but not in MCT injured lungs as compared to healthy controls (Figure 3B). Additionally, we observed discrepancies in Axin 1 mRNA expression level which was significantly upregulated in lungs 5 weeks after MCT injury, but its mRNA level was significantly reduced in MCT-PASMCs. Downregulation of upstream regulators of GSK3ß and GSK3ß itself (Figure S2), on mRNA level, in MCT-PASMCs as compared to healthy controls (Figure 3B) suggests dysregulation of canonical Wnt signaling pathway. Interestingly, further analysis of GSK3ß and its phosphorylated form at serine 9 residue in primary isolated pulmonary arterial smooth muscle cells (PASMCs) revealed an increase in total protein expression of GSK3ß and significant increase in its phosphorylated (inactivated) form in PASMCs isolated from MCT-PAH rats compared to control rats (Figure 3C, D).

### Growth factors signaling regulates GSK3ß phosphorylation in primary rat PASMCs

**Figure 4:**
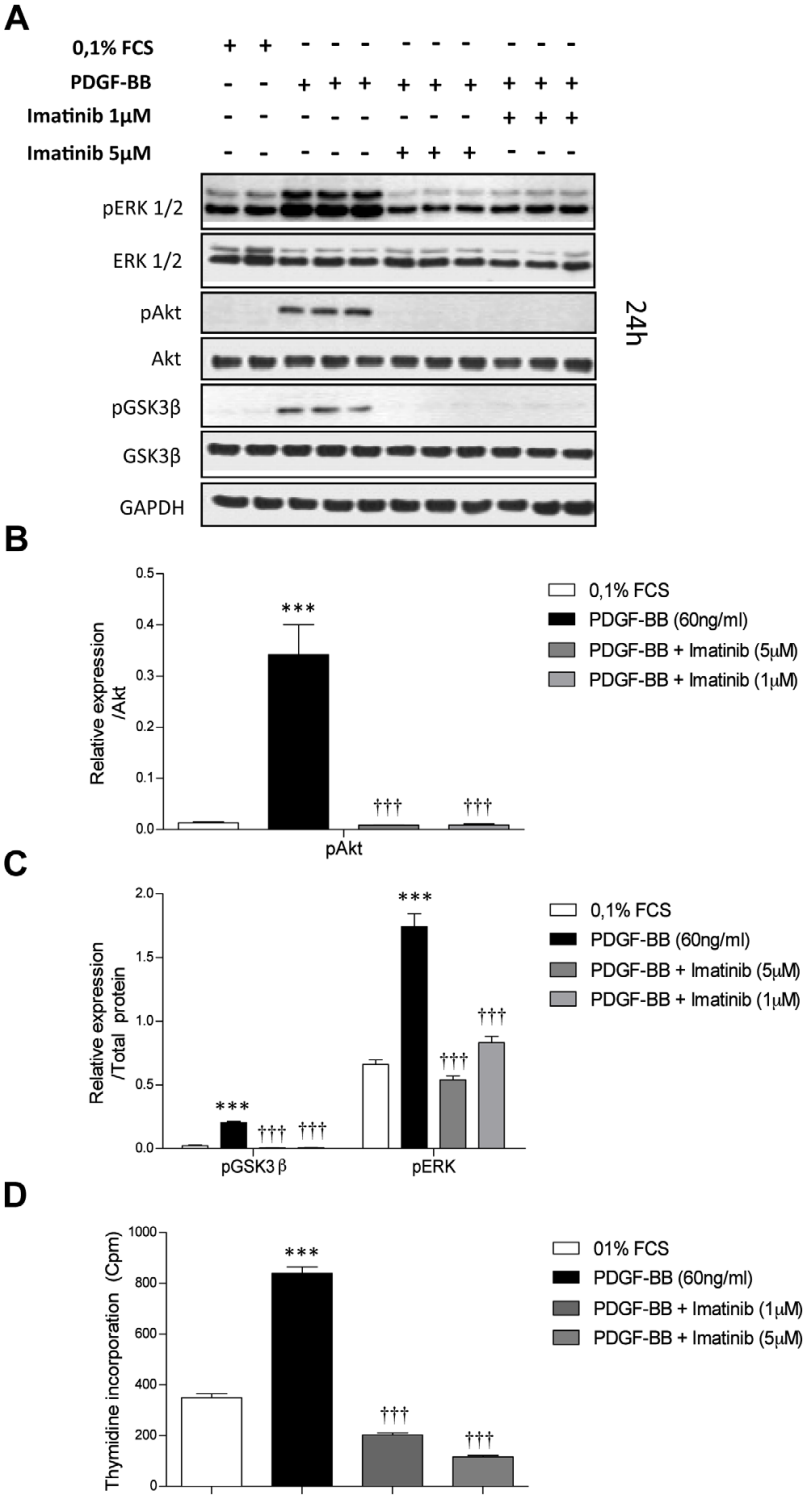
PDGF regulates GSK3β, Akt, ERK phosphorylation and proliferation in primary rat MCT-PASMCs. (**A**) Western blot analysis and subsequent (**B, C**) quantification of Akt, GSK3ß, ERK and phosphorylation status in primary rat MCT-PASMCs stimulated with PDGF-BB (60 ng/ml) alone or in combination with two doses of Imatinib (1 and 5 µM) for 24 hrs. GAPDH was used as reference loading control. (**D**) Proliferation of primary rat MCT-PASMCs was assessed by [3H]-thymidine incorporation (n = 6). Data were expressed as counts per minute (cpm) and normalized to the amount of cells per well. All values were expressed as mean ± SEM. Values were presented significant as *** P<0.001 *vs* control, ††† P<0.001 *vs* PDGF-BB.

**Figure 5:**
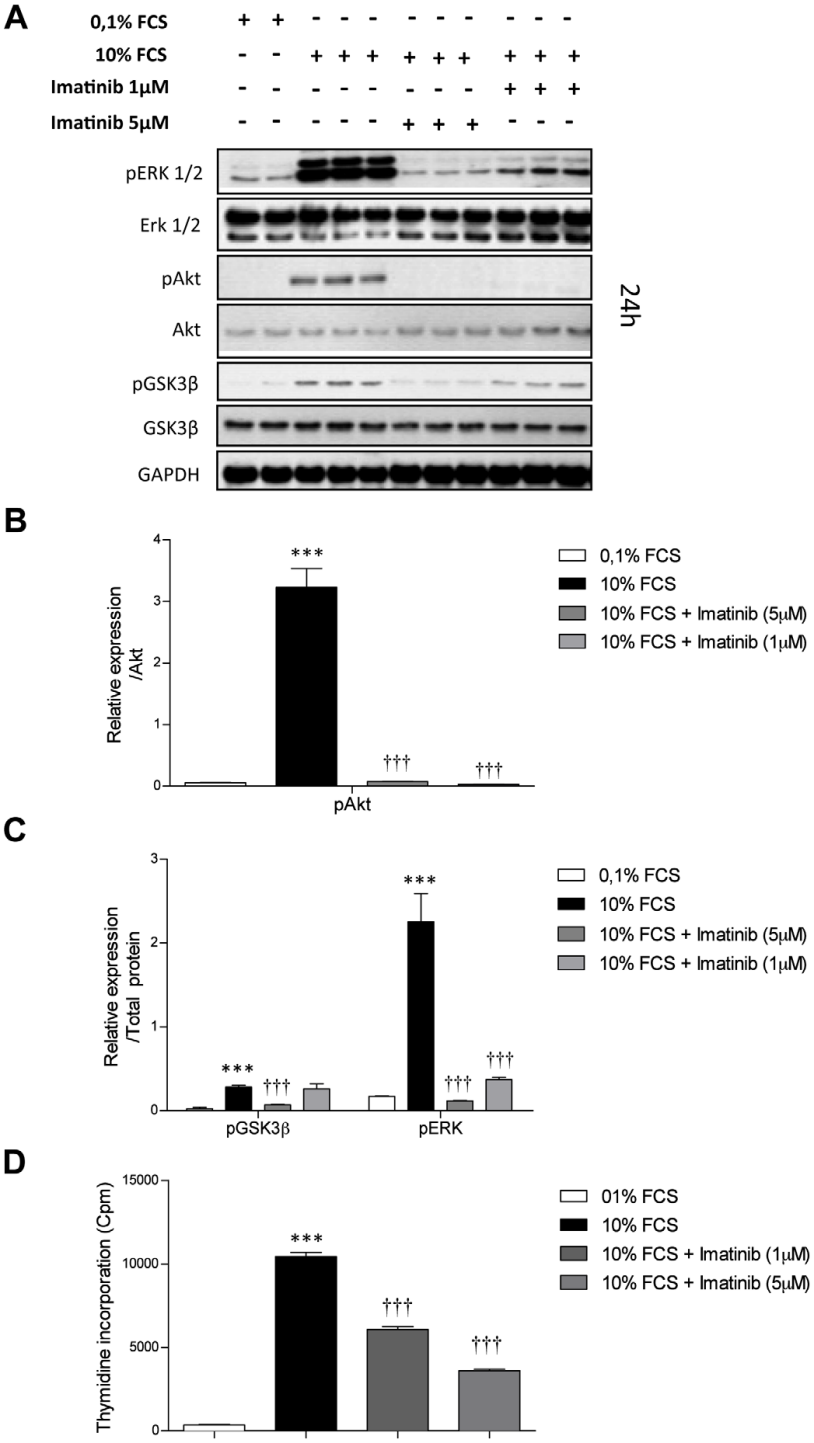
Serum regulates GSK3β, Akt, ERK phosphorylation and proliferation in primary rat MCT-PASMCs. (**A**) Western blot analysis and subsequent (**B, C**) quantification of Akt, GSK3ß, ERK and phosphorylation status in primary rat MCT-PASMCs stimulated with 10% FCS in combination with two doses of Imatinib (1 and 5 µM) for 24 hrs. GAPDH was used as reference loading control. (**D**) Proliferation of primary rat MCT-PASMCs was assessed by [^3^H]-thymidine incorporation (n = 6). Data were expressed as counts per minute (cpm) and normalized to the amount of cells per well. All values were expressed as mean ± SEM. Values were presented significant as *** P<0.001 *vs* control, ††† P<0.001 *vs* 10% FCS.

To investigate upstream regulators of GSK3ß, quiescent PASMCs were stimulated with PDGF-BB (60 ng/ml) for 6 (Figure S3) and 24 hrs (Figure 4) or 10% FCS (Figure 5) for 24 hrs. In addition, to verify the specificity of PDGF-BB mediated signaling, primary rat PASMCs were also simultaneously treated with Imatinib (1 and 5 µM). Proteins from untreated and stimulated cells were isolated and western blotting for AKT, GSK3ß, phospho-AKT and phospho-GSK3ß ser 9 and ERK and phospho-ERK were performed.

Interestingly, in all cases PDGF-BB and 10% FCS stimulation caused a significant increase in phospho-AKT and phospho-GSK3ß (Ser 9) in combination with significant activation of ERK (Figure 4A–C, 5A–C, Figure S3). The increased phosphorylation of AKT, GSK3ß at ser 9 site and ERK observed after stimulation with growth factors were significantly abrogated by Imatinib treatment (Figure 4A–C, 5A–C, Figure S3). Decreased phosphorylation status of AKT, GSK3β and ERK after Imatinib treatment were accompanied with significant decrease in proliferation capacity of these cells after 24 hrs stimulation with PDGF-BB as well as 10% FCS (Figure 4D, 5D). This suggest that GSK3β is a possible crucial player in growth factors signaling and abnormal proliferation of PASMCs in experimental PAH.

### Overexpression of GSK3β and phosphorylation deficiency of GSK3β influences PASMCs proliferation

To further evaluate the contribution of GSK3ß to vascular remodeling processes, we generated pcDNA 3.1 TOPO-cloning constructs carrying GSK3ß wild type (WT) cDNA and GSK3ß S9A (where serine at residue 9 is replaced to alanine) mutant. Sequence analysis performed on each construct confirmed the intended point mutations at specific sites in GSK3ß S9A (data not shown).

**Figure 6:**
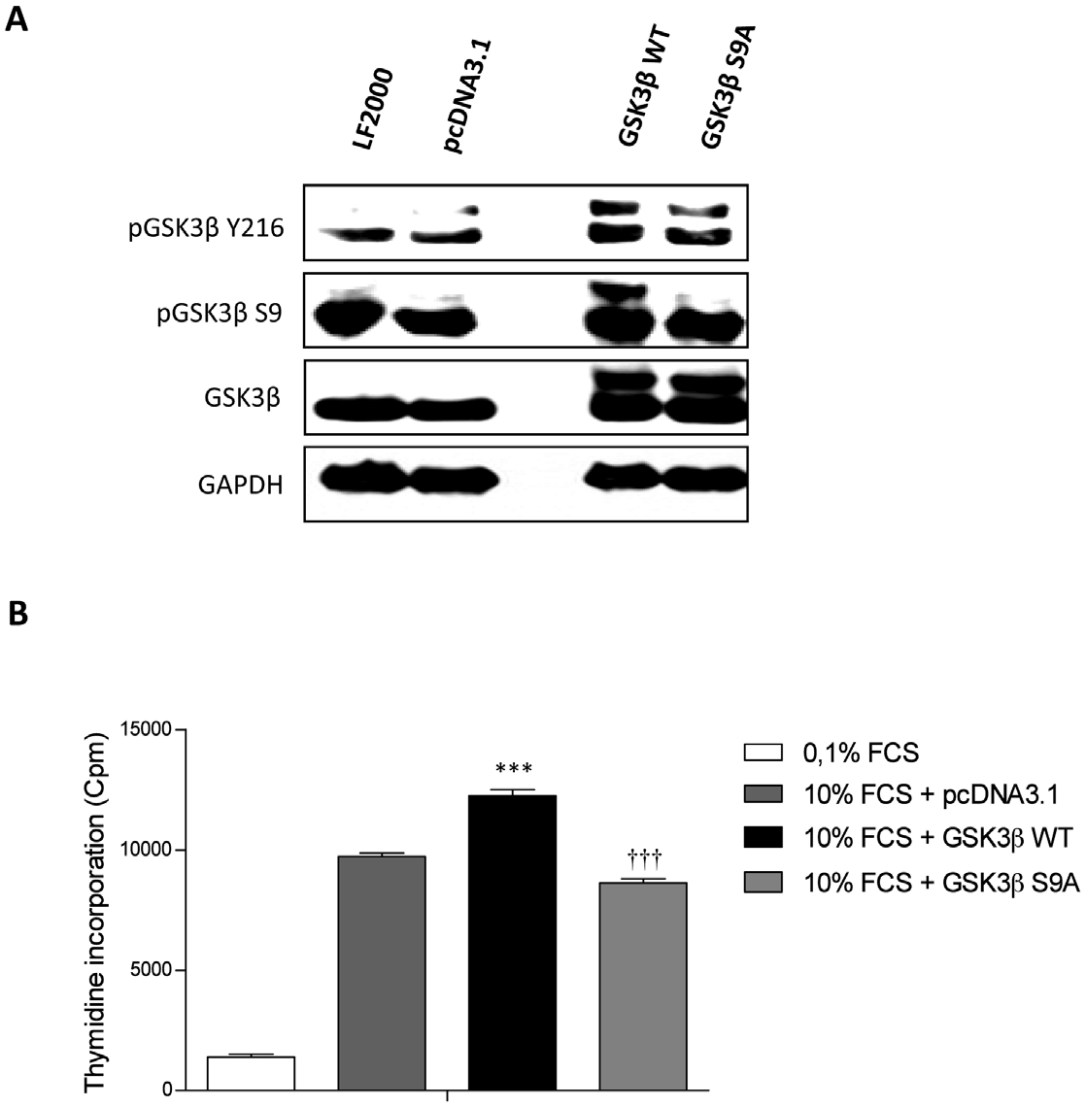
GSK3ß overexpression and S9A modification influences proliferation of primary rat PASMCs. (**A**) Primary rat PASMCs were transiently transfected with GSK3ß wild type (GSK3ß WT), constitutively active GSK3ß (GSK3ß S9A), empty vector (pcDNA3.1 TOPO). 24 hrs post transfection, expression and phosphorylation of GSK3ß were analyzed by western blotting. (**B**) Primary rat PASMCs transiently transfected with GSK3ß wild type (GSK3ß WT), constitutively active GSK3ß (GSK3ß S9A), empty vector (pcDNA3.1 TOPO) and proliferation was assessed after stimulation with 10% FCS for 24 hrs by [^3^H]-thymidine incorporation. All values are expressed as mean ± SEM (n = 6, WT or S9A n = 12). Data were expressed as counts per minute (cpm) and normalized to the amount of cells per well. Values were presented significant as ***P<0.001 *vs* 0.1% Empty Vector pcDNA3.1 and ††† p<0.001 *vs* GSK3ß WT transfected cells.

Transient overexpression of GSK3ß WT and GSK3ß S9A mutant in PASMCs resulted in a significantly enhancement of GSK3ß expression 24 hrs post transfection. Empty vector (EV) transfection caused no change in GSK3ß protein expression (Figure 6A). Importantly, serum stimulation did not cause GSK3ß phosphorylation in GSK3ß S9A mutant overexpressing cells while another tyrosine 216 residue was phosphorylated (Figure 6A). The gain of GSK3ß WT expression was coupled to a significantly increase of [^3^H]-thymidine uptake as compared to cells transfected with either empty pcDNA3.1 vector or Lipofectamine 2000 reagent (LF2000) only, 24 hrs post serum stimulation (Figure 6B). On the other hand, GSK3ß S9A mutant expression decreased [^3^H]-thymidine uptake, suggested that GSK3ß inactivation is involved in PASMCs proliferation (Figure 6B).

### Overexpression of GSK3β and phosphorylation deficiency of GSK3β influences proliferation via ERK phosphorylation

**Figure 7:**
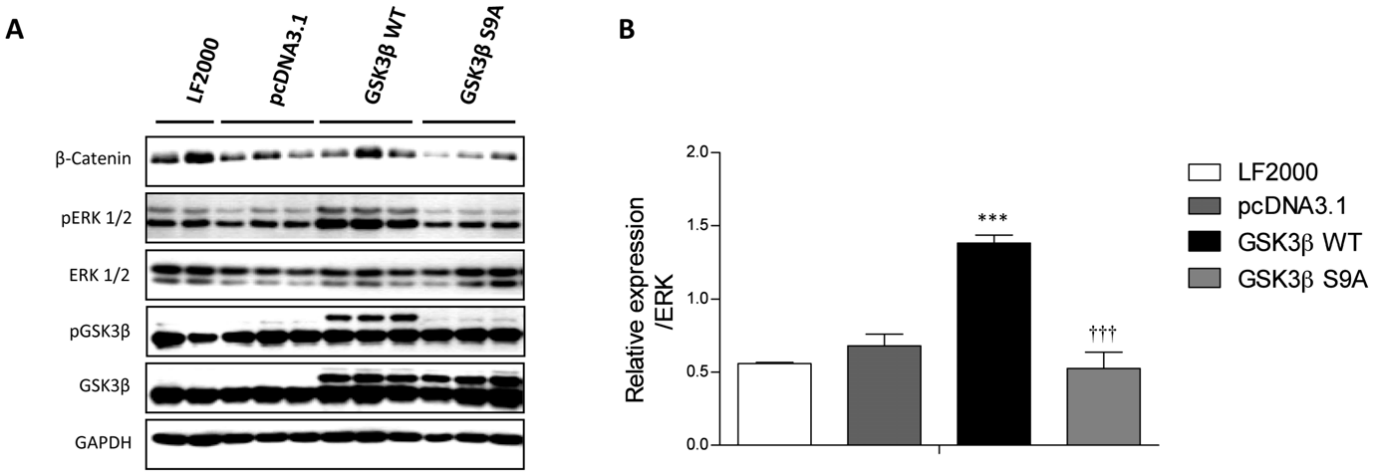
GSK3ß overexpression and S9A modification regulates ERK phosphorylation in primary rat PASMCs. (**A**) Primary rat PASMCs were transiently transfected with GSK3ß wild type (GSK3ß WT), constitutively active GSK3ß (GSK3ß S9A), empty vector (pcDNA3.1 TOPO). 24 hrs post transfection, expression and phosphorylation of GSK3ß and ERK were analyzed by western blotting followed by (**B**) densitometric quantification. All values are expressed as mean ± SEM (n = 4). Values were presented significant as ***P<0.001 *vs* 0.1% Empty Vector pcDNA3.1 and ††† p<0.001 *vs* GSK3ß WT transfected cells.

Transient overexpression of GSK3ß WT significantly increased ERK phosphorylation compared to empty pcDNA3.1 vector transfection (Figure 7A, B). In contrary, GSK3ß S9A mutant decreased ERK1/2 phosphorylation, in serum induced proliferation of PASMCs (Figure 7A, B).

### Increase in GSK3β protein levels in human PAH lung explants

**Figure 8:**
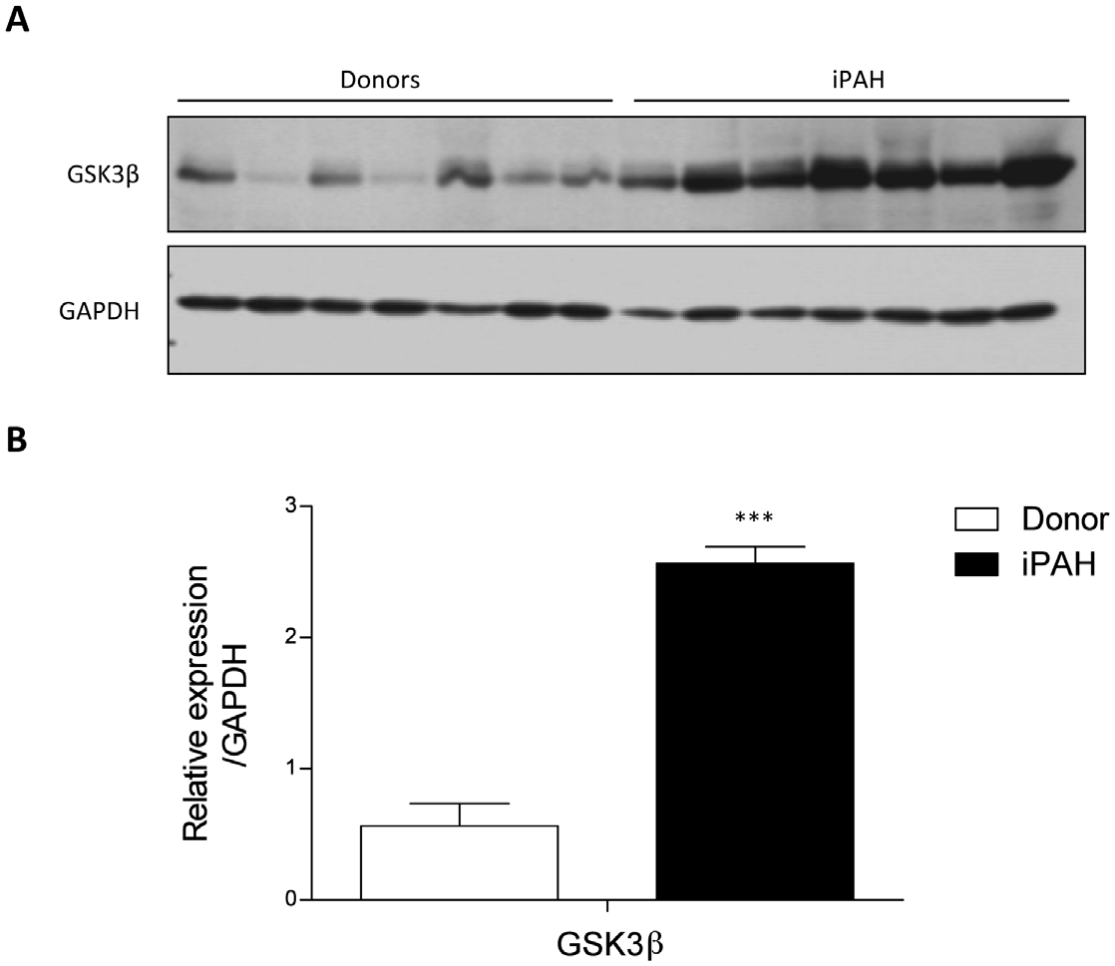
Increased GSK3ß and its phosphorylated form in human lungs of healthy donor and iPAH patients. (**A**) Protein expression as analyzed by western blotting and subsequent (**B**) densitometric quantification of GSK3ß and in human lungs of healthy donor and iPAH patients. GAPDH was used as a loading control. Values were presented significant as ***P<0.001 *vs* control lungs. All values were expressed as mean ± SEM (n = 7).

Expression of GSK3ß was investigated also in iPAH patients lung explants. While mRNA levels of GSK3ß was not regulated in iPAH patient lungs (Figure S4), western blotting analysis showed significant upregulation of total GSK3ß protein in iPAH lung homogenates compared to healthy donor lung homogenates (Figure 8A, B).

## Discussion

This study has 4 salient features. First, GSK3ß and the phosphorylated form of GSK3ß (inactivation) are increased in MCT lungs and PASMCs isolated from monocrotaline induced pulmonary arterial hypertensive rats compared to control rats. Second, both PDGF and FCS stimulation induced GSK3ß phosphorylation (inactivation) in PASMCs. Third, treatment with the PDGFR inhibitor, Imatinib, attenuated growth factors-induced GSK3ß and ERK phosphorylation. Fourth, overexpression of wild type and constitutively activate form of GSK3ß (GSK3ß S9A; 9th serine replaced to alanine) influenced serum induced MCT-PASMCs proliferation by regulating ERK phosphorylation. This study supports a central role for GSK3ß in vascular remodeling processes and suggests a novel therapeutic opportunity for the treatment of pulmonary hypertension.

The dynamic process of pulmonary vascular remodeling involves numerous molecular signaling cascades governing PASMCs proliferation, differentiation and migration [4], [29]. We hypothesized that GSK3ß signaling plays a critical regulatory role in pulmonary vascular remodeling. In accord with our hypothesis, we demonstrated significant increase in total GSK3ß expression levels as well as its phosphorylation status in lungs and PASMCs in response to MCT injury and total GSK3ß in human iPAH lung explants. Interestingly, the increase in GSK3ß and its phosphorylated form, observed in primary PASMCs isolated from MCT-PAH rats as well as in rat and human lungs, suggest that aberrant GSK3ß signaling may trigger the proliferative phenotype of PASMCs. In line with this notion, aberrant GSK3ß signaling was recently implicated in various vascular- and fibro-proliferative diseases [23], [30], [31].

GSK3ß remains increased and inactivated 5 weeks after MCT injury, which is accompanied by decreases in mRNA expression of canonical Wnt ligands (Wnt1 and Wnt3a) and an increase in non-canonical Wnt5a ligand, 3 and 5 weeks after MCT injury. However, GSK3ß was downregulated at the mRNA level in MCT-PASMCs compared to control-PASMCs. We assume that downregulation of GSK3ß mRNA levels concurrent with upregulation of protein expression may occur when a protein's half-life is increased due to stabilization-components involved with the protein's normal turnover may be disrupted. It is also possible that the protein may become stabilized through protein-protein interactions. Additionally, this can be explained by transcriptional repression when high levels of proteins accumulate. Furthermore as delineated in our study, GSK3ß phosphorylation is regulated by growth factors, i.e. PDGF-BB that were highly upregulated in PAH. Hence we believe that GSK3ß is clearly dependent on multiple lines of regulation in addition to the phosphorylation state of GSK3ß [32], [33], [34].

In our study, upregulation of non-canonical [35], [36] Wnt5a mRNA in MCT-lungs is in line with previously published data, showing involvement of non-canonical Wnt signaling in human IPAH [37]. Moreover, downstream targets of the non-canonical Wnt pathway, like Rho-kinases or calcium signaling, were shown to significantly contribute to PASMCs proliferation and vasocontriction and demonstrated therapeutic potential in pulmonary hypertension [38], [39], [40].

Our data indicate that MCT-PASMCs are hyper-proliferative in the presence of FCS and PDGF compared to control-PASMCs. Interestingly, our results showed that PDGF and FCS stimulation on MCT-PASMCs, acting via PI3-kinase-dependent activation of AKT, causes GSK3ß (Ser 9) phosphorylation and GSK3ß inactivation followed by ERK activation, which is potentially suppressed by Imatinib. A similar role of growth factors, such as PDGF, IGF and EGF, mediated GSK3ß inactivation was described previously [41], [42]. Furthermore, several kinases were also shown to be capable of mediating ser 9 phosphorylation, including AKT, PKA, PKC and Wnts [16], [32], [43]. Considering the crucial role of PDGF signaling in pulmonary vascular remodeling [8], [9], the increased presence of growth factors signaling in human and experimental PAH [8], [10], [13] and PDGF and FCS mediated alteration of GSK3ß activity, collectively suggests GSK3ß plays an important role in the pathogenesis of PAH.

The effects of GSK3ß are also regulated by Wnt signaling pathway protein complex formation, a process involved in modulating ß-Catenin levels [18], [44]. Future studies are needed to study the regulation of canonical Wnt signaling and the multitude of factors regulating ß-Catenin expression in the pathogenesis of pulmonary hypertension. Albeit recent studies suggest that recruitment of both canonical and non-canonical Wnt pathways promote pulmonary arterial endothelial cell proliferation, survival, and migration. In addition, it was demonstrated that both canonical and non-canonical Wnt pathways are required for BMP-2-mediated angiogenesis in severe combined immunodeficient (SCID) mice [45]. These findings may help better understand the pathogenesis of pulmonary hypertension, a disease that featured with the loss of small precapillary arteries.

In the present study, overexpression of GSK3ß significantly increased expression of GSK3ß that was accompanied by increased proliferation capacity of MCT-PASMCs proliferation after FCS stimulation. Constitutive activation of GSK3ß significantly reduced expression of phospho-GSK3ß and PASMCs proliferation. This effect can be due to GSK3ß phosphorylation of a diverse group of substrates or by inhibition of transcription factor activation such as p53, CREB and ß-Catenin [17], [18], [19]. In addition, GSK3ß constitutively activation (S9A) has also been shown to directly effects cyclin D1 expression, independent of ß-Catenin [46]. Here, we show that overexpression of wild-type GSK3ß significantly influenced the proliferation capacity of MCT-PASMCs via regulating phosphorylation of ERK. In a recent study by Wang *et al.*, it was shown that GSK3ß acts as a negative regulator of ERK in human colon cancer cells [47]. Our study shows that constitutive activation of GSK3ß significantly reduced phospho-GSK3ß levels and PASMCs proliferation that was accompanied by a significant decrease in ERK phosphorylation. These results collectively suggest that modification of GSK3ß can significantly influence highly dysregulated growth factors signaling associated with abnormal proliferation of PASMCs in PAH.

Although mRNA levels of GSK3ß is downregulated in MCT-PASMCs and in human iPAH patient lungs, along with other canonical Wnt signaling dependent genes we found that protein levels of GSK3ß increased in a time dependent fashion with disease progression in the MCT-induced PAH model (lungs and MCT-PASMCs) and also in explanted iPAH patient lungs, suggesting a role for GSK3ß in disease progression, potentially independent of canonical Wnt signaling. Recently a crucial role for GSK3ß in systemic vascular remodeling was reported [48], [49]. Authors demonstrated for the first time *in vivo* that active GSK3ß gene transfer results in a significant reduction in neointima formation in the restenosis model of balloon injury in rat carotid arteries. These effects were attributable, at least in part to the ability of GSK3ß to inhibit smooth muscle proliferation and to promote sustained apoptosis. This concept was extended to demonstrate that GSK3ß plays a significant role in VSMCs proliferation and apoptosis in vascular remodeling after balloon injury [49]. Similarly, in our study the inactivation of GSK3β by serine 9 phosphorylation was observed 5 weeks after MCT injury in rats, both in PASMCs and lung homogenates. This suggest that the introduction of active GSK3β (S9A) may also prove beneficial for regression of vascular remodeling in experimental PAH.

To our knowledge this is the first study to demonstrate that GSK3ß is significantly altered in the pathogenesis of experimental as well as human PAH and the regulatory role for GSK3ß in pulmonary arterial smooth muscle cell proliferation. This study supports a central role for GSK3ß in vascular remodeling processes and suggests a novel therapeutic opportunity for the treatment of pulmonary arterial hypertension.

## Supporting Information

Figure S1 **MCT-PASMCs display significant increase of PDGF-BB-induced proliferation capacity as compared to healthy control-PASMCs.** Proliferation capacity of primary rat MCT-PASMCs compared to healthy control PASMCs isolated from rat lungs 5 weeks post MCT injury in 10% FCS conditioned media was assessed by [3H]-thymidine incorporation (n = 5). Data were obtained as counts per minute (cpm) and normalized to the amount of cells per well. All values were expressed as percentage of proliferation capacity (mean ± SEM). Values were presented significant as *** P<0.001 *vs* control.
(TIF)

Figure S2 **Expression of GSK3β in rat MCT-PASMCs.** mRNA expression of GSK3β in MCT-PASMCs after 5 weeks of MCT-induced PAH rats, as analyzed by quantitative real-time PCR. All values were given as the mean ± SEM (n = 3) and were normalized to Porphobilinogen deaminase (PBGD). Values were presented significant as *P<0.05, *vs* PASMCs isolated from healthy rat lungs. Healthy controls were set as 1 on X axis and expression profile from 5 weeks MCT-PASMCs were presented as fold of gene regulation.
(TIF)

Figure S3 **PDGF regulates GSK3β, Akt and ERK phosphorylation in primary rat MCT-PASMCs.** (**A**) Western blot analysis and subsequent (**B, C**) quantification of Akt, GSK3ß and ERK phosphorylation status in primary rat MCT-PASMCs stimulated with PDGF-BB (60 ng/ml) alone or in combination with two doses of Imatinib (1 and 5 µM) for 6 hrs. All values were expressed as mean ± SEM (n = 4). Values were presented significant as *** P<0.001 *vs* control, ††† P<0.001 *vs* PDGF-BB. GAPDH was used as reference loading control.
(TIF)

Figure S4 **GSK3β is not significantly regulated in iPAH patient lungs on mRNA level.** mRNA expression of GSK3ß in donor lungs and iPAH patient lungs as analyzed by quantitative real-time PCR. All values were normalized to Porphobilinogen deaminase (PBGD) and determined as fold of gene regulation. All values were expressed as mean ± SEM (n = 8).
(TIF)
