# Supplementary figures and images for: Glycogen Synthase Kinase 3beta Contributes to Proliferation of Arterial Smooth Muscle Cells in Pulmonary Hypertension
Source: PLoS One. 2011 Apr 18;6(4):e18883. doi: 10.1371/journal.pone.0018883 (PMC3078925; doi:10.1371/journal.pone.0018883)

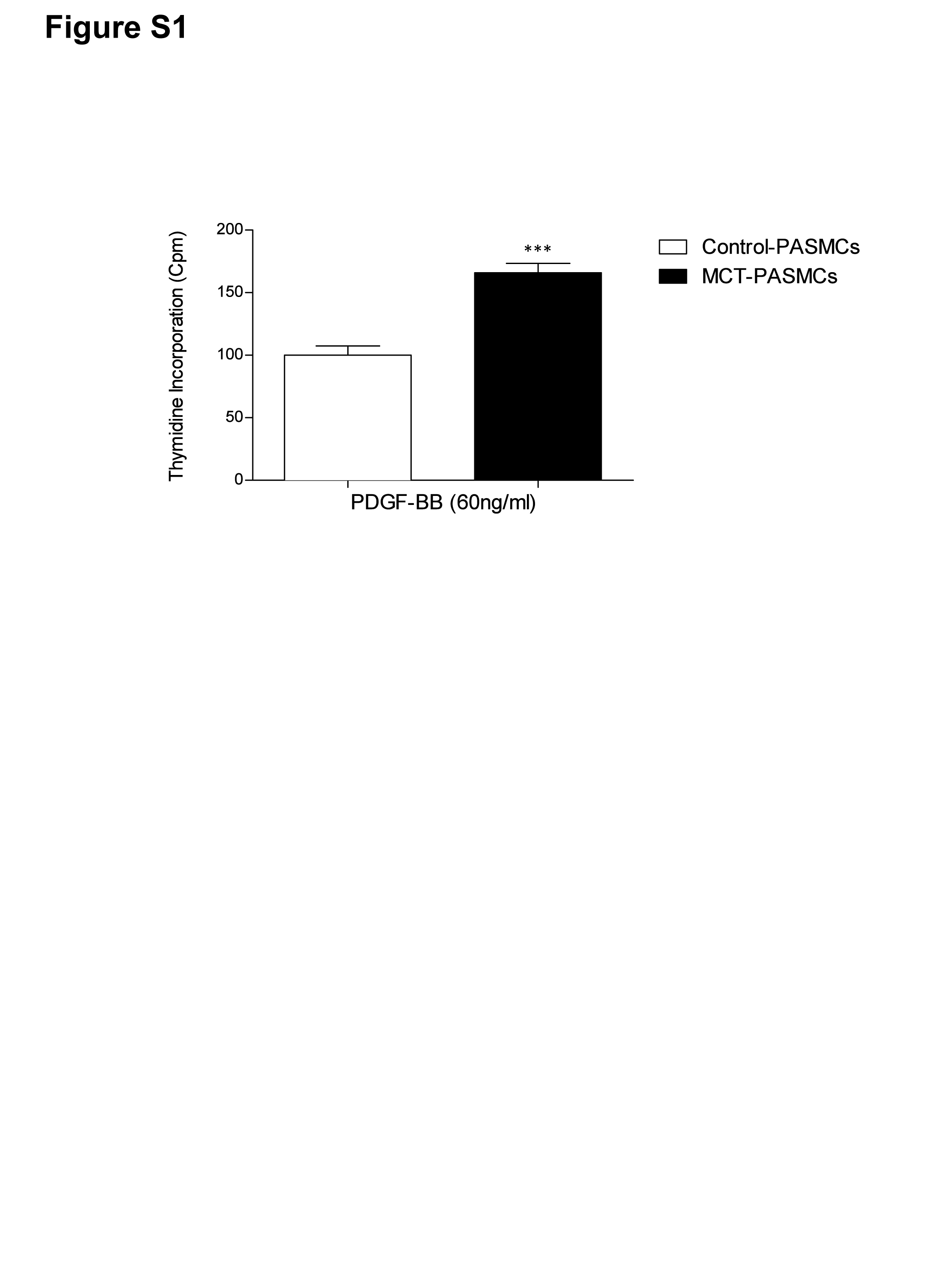

Supplement: Figure S1 — MCT-PASMCs display significant increase of PDGF-BB-induced proliferation capacity as compared to healthy control-PASMCs. Proliferation capacity of primary rat MCT-PASMCs compared to healthy control PASMCs isolated from rat lungs 5 weeks post MCT injury in 10% FCS conditioned media was assessed by [3H]-thymidine incorporation (n = 5). Data were obtained as counts per minute (cpm) and normalized to the amount of cells per well. All values were expressed as percentage of proliferation capacity (mean ± SEM). Values were presented significant as *** P<0.001 vs control. (TIF) [file pone.0018883.s001.tif]

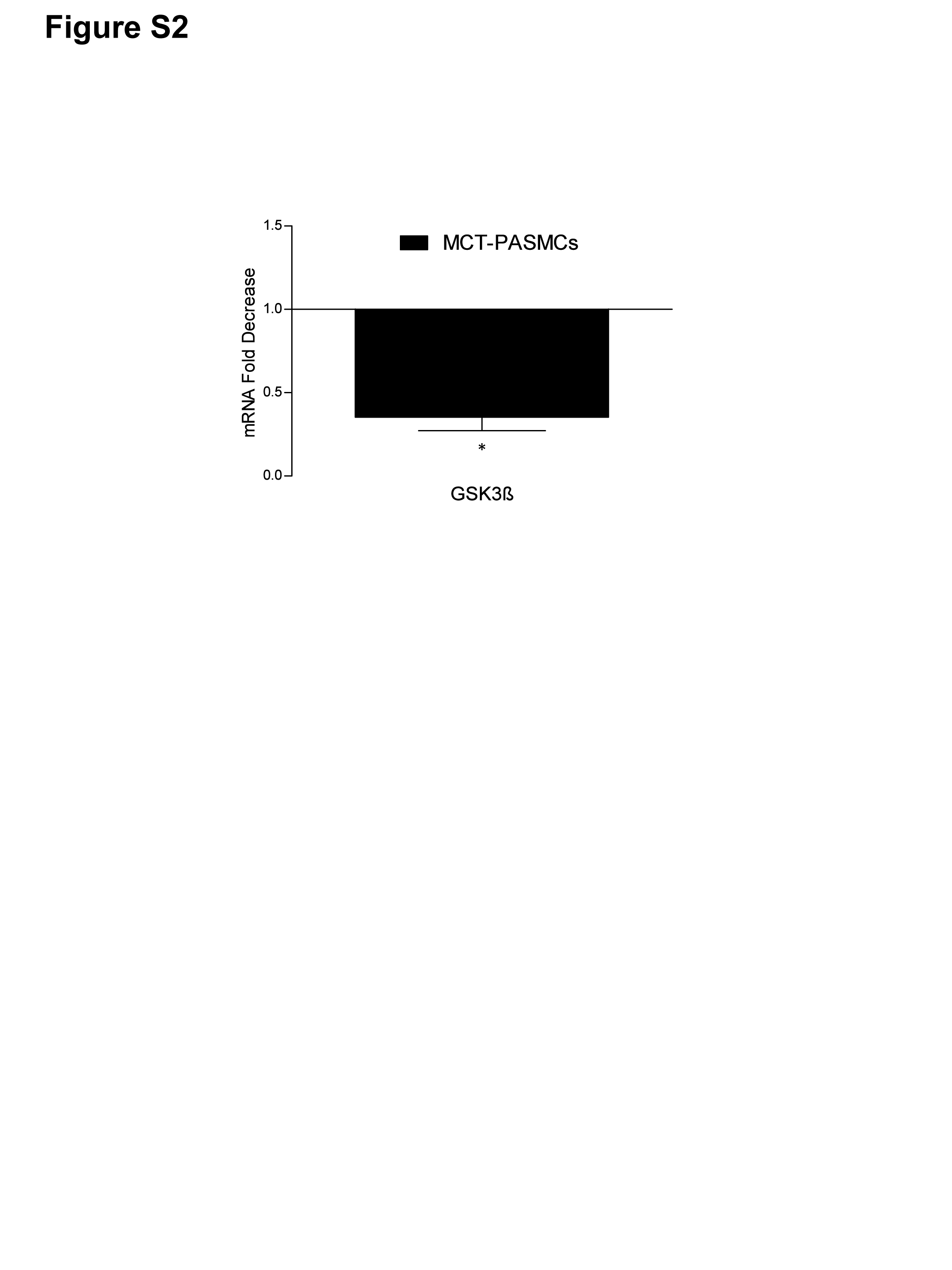

Supplement: Figure S2 — Expression of GSK3β in rat MCT-PASMCs. mRNA expression of GSK3β in MCT-PASMCs after 5 weeks of MCT-induced PAH rats, as analyzed by quantitative real-time PCR. All values were given as the mean ± SEM (n = 3) and were normalized to Porphobilinogen deaminase (PBGD). Values were presented significant as *P<0.05, vs PASMCs isolated from healthy rat lungs. Healthy controls were set as 1 on X axis and expression profile from 5 weeks MCT-PASMCs were presented as fold of gene regulation. (TIF) [file pone.0018883.s002.tif]

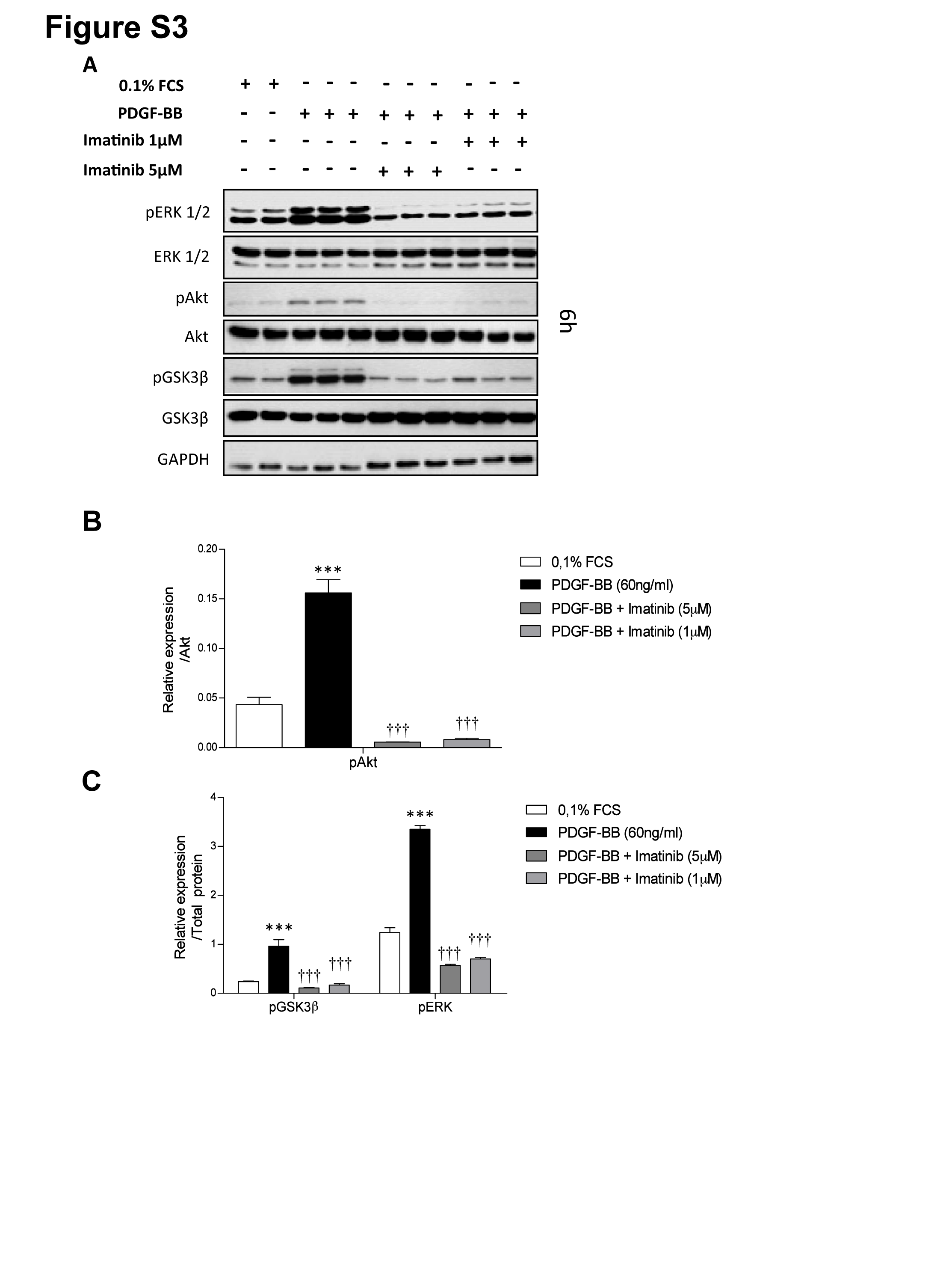

Supplement: Figure S3 — PDGF regulates GSK3β, Akt and ERK phosphorylation in primary rat MCT-PASMCs. (A) Western blot analysis and subsequent (B, C) quantification of Akt, GSK3ß and ERK phosphorylation status in primary rat MCT-PASMCs stimulated with PDGF-BB (60 ng/ml) alone or in combination with two doses of Imatinib (1 and 5 µM) for 6 hrs. All values were expressed as mean ± SEM (n = 4). Values were presented significant as *** P<0.001 vs control, ††† P<0.001 vs PDGF-BB. GAPDH was used as reference loading control. (TIF) [file pone.0018883.s003.tif]

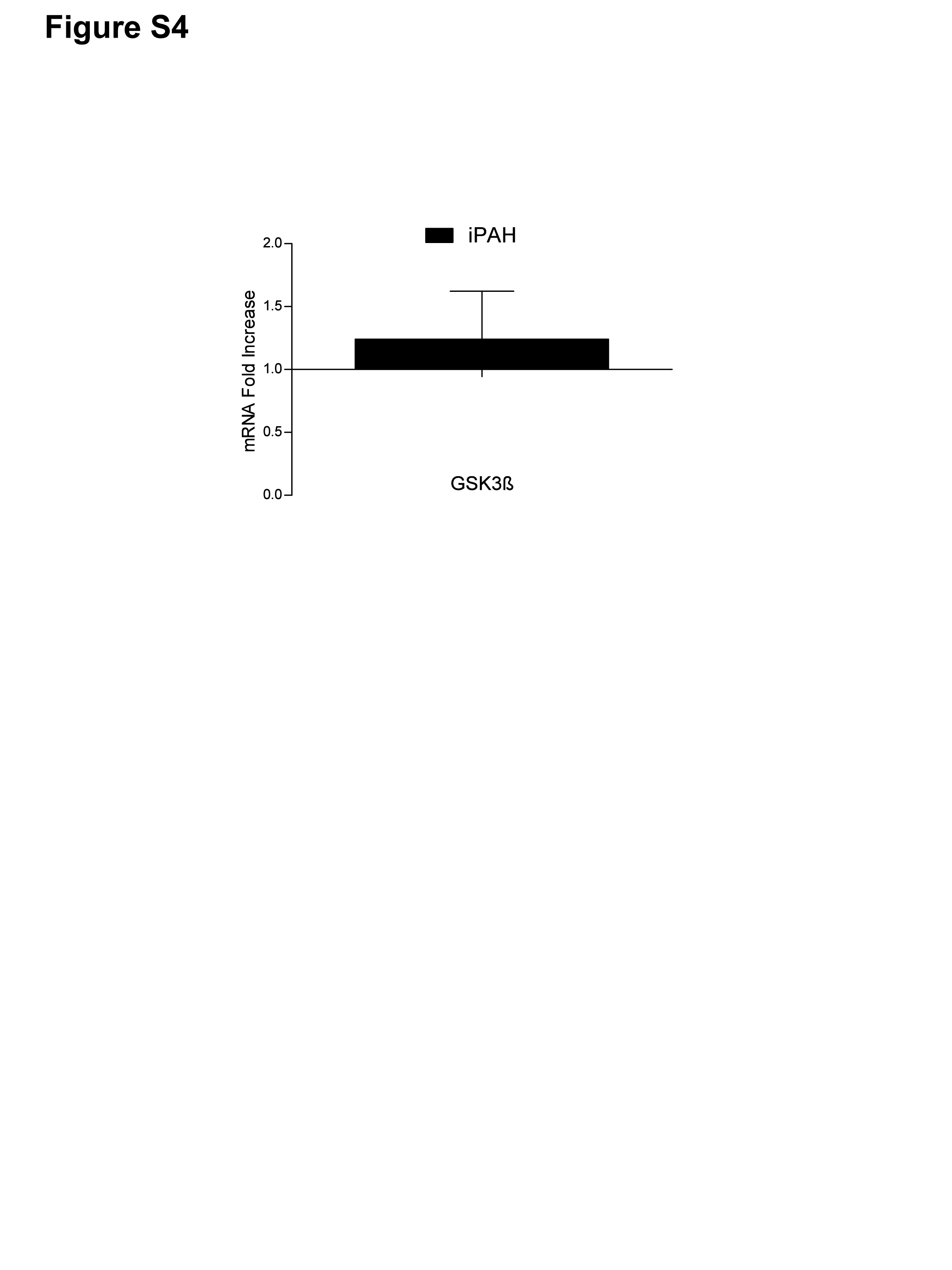

Supplement: Figure S4 — GSK3β is not significantly regulated in iPAH patient lungs on mRNA level. mRNA expression of GSK3ß in donor lungs and iPAH patient lungs as analyzed by quantitative real-time PCR. All values were normalized to Porphobilinogen deaminase (PBGD) and determined as fold of gene regulation. All values were expressed as mean ± SEM (n = 8). (TIF) [file pone.0018883.s004.tif]
